# Regional morphological adaptations of vastus lateralis muscle in response to different progressive resistance training programs: A randomised controlled trial

**DOI:** 10.17159/2078-516X/2024/v36i1a18549

**Published:** 2024-09-15

**Authors:** R Longrak, W Sonchan, W Jaidee

**Affiliations:** 1 Faculty of Sport Science, Burapha University, Chon Buri, Thailand; 2 Faculty of Medicine, Burapha University, Chon Buri, Thailand

**Keywords:** Morphology, hypertrophy, muscle area, muscle thickness, fascia thickness

## Abstract

**Background:**

Resistance training often increases muscle size, a phenomenon known as muscle hypertrophy. These morphological adaptations were typically documented to occur in a non-uniform pattern. Investigating the specific morphological adaptations to different training programs was of interest.

**Objectives:**

This study aimed to investigate two resistance training programs, a high-intensity program (HI) and a combined high-intensity with low-intensity blood flow restriction program (MIX), on morphological adaptations of vastus lateralis muscle in healthy young men.

**Methods:**

Eighteen active participants were recruited and randomly assigned to the HI (n = 10) or MIX (n = 8) groups, undergoing different 6-week resistance training programs. The training volume set was equated and progressively increased from three sets in weeks 1 and 2 to six sets, and eight sets in weeks 3–4 and 5–6, respectively. Three specific regions of vastus lateralis were assessed by magnetic resonance imaging (MRI) and ultrasound imaging (US) during pre-and post-intervention.

**Results:**

Statistical analysis revealed statistically significant increases in muscle area at the proximal (HI: Δ12%, MIX: Δ9.2%), middle (HI: Δ8.7%, MIX: Δ9.0%), and distal (HI: Δ14%, MIX: Δ13%) regions. Additionally, both HI and MIX groups showed statistically significant increases in the sum of muscle thickness post-intervention (HI: Δ12%, MIX: Δ19%) and in the sum of fascia thickness post-intervention (HI: Δ27%, MIX: Δ54%). Despite the MIX group training with higher volume load, no statistical differences were observed between groups for any week.

**Conclusion:**

These findings suggested that both HI and MIX programs effectively induced increases in muscle area and sums of muscle and fascia thickness in healthy young men, allowing practitioners to choose either program based on individual preferences and constraints.

Resistance training has always been fundamental in exercise physiology, providing a powerful means to enhance muscle ability to produce force and mass.[1] This form of exercise involves the utilisation of various resistance modalities, such as free weights, pin-load machines, and bodyweight exercises, to disrupt homeostasis within the musculature, leading to physiological and morphological adaptation.[2] For example, one of the primary objectives of resistance training is to promote muscle hypertrophy, characterised by an increase in muscle size due to the enlargement of individual muscle fibres resulting from increased muscle protein synthesis.[3] For example, previous studies demonstrate that muscle thickness and areas of various muscles such as triceps brachii, biceps brachii, pectoralis major, biceps femoris, as well as vastus lateralis significantly increased after a period of resistance training.[4–6]

As research on resistance training progressed, alternative training methodologies were continually explored to meet various specific training goals, such as increasing strength, improving muscle endurance, and enhancing muscle size. One such emerging approach was resistance training with blood flow restriction (BFR), also known as occlusion training.[7] This innovative technique involved applying external pressure from the cuffs to the proximal limbs during exercise, which restricted venous return while partially allowing arterial flow, creating a hypoxic environment within the working muscles.[7] This approach was beneficial because it allowed individuals unable to lift heavy weights, such as injured athletes or untrained individuals, to still achieve muscle growth albeit with lower external loads. Additionally, BFR could reduce joint strain, enhance muscle fiber recruitment and growth hormone secretion, cause metabolic stress and cell swelling, and potentially trigger muscle hypertrophy.[8] For instance, Ellefsen and team found that low-intensity resistance training at only 30% of repetition maximum (1RM) with BFR resulted in a significant increase of quadriceps muscle area by approximately 6% after 12 weeks of training.[4]

Structuring the optimal resistance training program has always been of interest to researchers. Numerous previous studies have attempted to introduce novel training methods to achieve better results in hypertrophy. For example, Stragier and colleagues proposed that the *3/7 method* was superior to a traditional eight sets of six repetitions training program in inducing muscle thickness of the biceps brachii.[9] In contrast, Amirthalingam and team demonstrated that the *German volume training 10×10 method* was not more effective than a traditional five sets of ten repetitions after six weeks of training for increasing arms, legs, and trunk hypertrophy.[10] Therefore, the current study aims to investigate two different resistance training programs: *the high-intensity program (HI)* and the *combined high-intensity with low-intensity blood flow restriction program* (MIX). Specifically, the study assesses their effects on muscle area, muscle thickness, and fascia thickness of the vastus lateralis muscle.

## Methods

### Study design

The study aimed to compare regional morphological adaptations of two different training programs: HI and MIX. Employing a single-blind experimental design, two experimental groups were incorporated to address the research questions, with standardised controls of training factors such as volume set, exercise order, execution pattern, repetition tempo, and rest interval time. The eligibility criteria restricted participation to individuals who had not engaged in structured resistance training in the last six months prior to the start of the study, aiming to minimize experience-related biases. Resistance training was conducted in a laboratory setting, supervised by a certified personal trainer. Baseline testing, including MRI for muscle area and US for muscle and fascia thickness. Post-intervention measurements were conducted one week after the final training session.

### Ethical approval

Ethical approval for this study was granted by the Burapha University Ethics Committee (Code: G-HS046/2566(C1)). Prior to participation, all participants were informed about the risks and benefits of the study and provided with a signed consent form in accordance with the Helsinki Declaration.

### Participants

Twenty healthy young men, active in various sports such as basketball, cycling, and football, were recruited for this study. The sample size was determined by using the G*Power software (version 3.1.9.7). The input parameter was Effect Size 0.75, α err prob 0.05, Power 0.80, with 20% dropout rate allowed. None had participated in a structured strengthening program in the last six months. Recruitment took place at the Faculty of Sports Science, Burapha University. Those currently undergoing resistance training were ineligible. All participants met specific inclusion criteria: no functional limitations affecting strength tests or training protocols, no history of using pharmacological substances, ergogenic drugs and any performance supplements, or anabolic steroids that could influence muscle thickness, and they underwent a health assessment by a physician.

### Resistance training programs

#### High intensity program (HI)

**Fig. 1:**
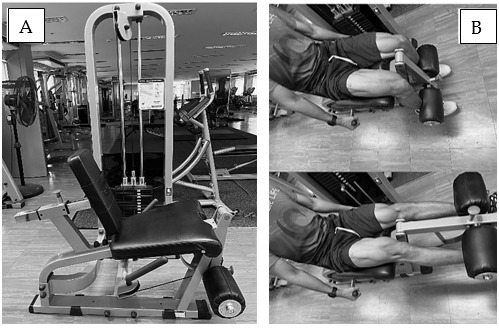
(A) Knee extension machine (Body-Solid, USA). (B, upper) Starting position and finishing position of eccentric phase. (B, lower) finishing position of concentric phase of knee extension exercise

HI program was structured to last for six weeks, with 1 training session per week. In the first two weeks, each training session consisted of three sets of knee extension exercise, with a knee extension machine (Body-Solid, USA) (Figure 1A). The training intensity was set at 70% 1RM, aligning with the American College of sports medicine’s guidelines for promoting muscular hypertrophy in novice to intermediate individuals.[1] All training sets were carried out to failure. This method guarantees that most of the muscle fibres of high threshold motor units are recruited to get under tension.[14] The repetition tempo was fixed at two seconds for eccentric and concentric phases. The resting period was 60 seconds between each set to minimise accumulated fatigue and enable higher training volumes in subsequent sets.[11] The set was terminated when the full range of motion of exercise could not be conducted. In the third and fourth weeks, the training volume set was progressively doubled; in the fifth and sixth weeks, the set was increased again to eight sets (Figure 1B).

#### High intensity with low-intensity blood flow restriction program (MIX)

For the MIX group, in the first two weeks, participants performed two high-intensity sets at 70% 1RM and a single low-intensity set at 30% 1RM with a practical BFR technique of similar knee extension exercises. Practical BFR was applied using elastic wraps (GRIZZLY FITNESS, USA). The wraps were fastened around the upper limbs proximally, around the insertion of shoulder muscles just distal to the deltoid tuberosity of the humerus. For the lower limbs, the wraps were applied at the proximal thigh, just distal to the greater trochanter of the femur. The pressure was adjusted to exceed 40% of the perceived arterial occlusion pressure (AOP). This threshold of 40% AOP was the lowest yet most effective AOP for inducing hypertrophy when utilising the BFR technique, according to the literature.[12] Before the study began, each participant was initially instructed and familiarised with AOP using a pneumatic cuff (H+CUFF, USA). They were fitted with a pneumatic cuff and exposed to the lowest pressure that completely occluded arterial blood flow. The pressure was gradually increased by 10–20 mmHg to determine their 100% AOP, verified by a vascular Doppler. Subsequently, they were subjected to 40% of their AOP on both upper and lower extremities, with the pressure alternated on and off at a 15:30 second ratio. Once comfortable, participants used elastic wraps to apply a similar perceived 40% AOP pressure and were encouraged to fasten the wraps slightly tighter to ensure the pressure exceeded 40%. In the third and fourth week, three sets of high intensity and three sets of low intensity with practical BFR were performed. In the fifth and sixth week, the training volume set progressed to four sets of high intensity and four sets of low intensity with practical BFR. Similarly to the HI group, all training variables were same.

### Muscle area, muscle thickness, and fascia thickness measurements

**Fig. 2:**
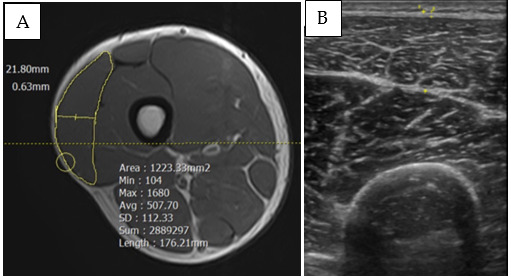
Example images of (A) muscle area and (B) muscle thickness of vastus lateralis of a random participant

The muscle areas were assessed by magnetic resonance imaging (MRI) using a 1.5-T scanner (Vantage Elan, Canon, USA), which included standard axial T1-weighted and T2-weighted fat-saturated sequences, as well as coronal T1-weighted and T2-weighted fat-saturated sequences (Figure 2A). Muscle measurements were taken at the proximal region 30%, middle region 50% and distal region 70% of the femur length as identified from the greater trochanter to the lateral epicondyle of the femur. Hypertrophy was measured at three regions as hypertrophy often occurs non-uniformly. Thus, summarising hypertrophic results from a single region might not accurately represent the overall adaptation from the training program.[13]

Similarly, muscle thickness and fascia thickness of vastus lateralis muscle were assessed by ultrasound (US) imaging. The B-mode US device (LOGIQ E10 Series, GE Healthcare, USA) equipped with a curvilinear probe was used. Muscle thickness was determined as the linear distance between the deep and superficial aponeuroses of the muscle, utilising a frequency of 5–10MHz (Figure 2B). The thickness was measured at three specific points along the femur similarly to MRI. Additionally, the fascia thickness, as superficial fascia, was collected at similar points of muscle thickness to determine the fascia remodelling characteristics of each training program.

### Statistical analyses

The statistical analysis was calculated to investigate morphological changes after two different training programs. The Shapiro-Wilk test was analysed for the distribution of data. Descriptive statistics were used to summarise baseline anthropometrics and morphologies in both groups. Baseline variables between groups was compared by One-way ANOVA. Levene’s test was used to assess the homogeneity of variances. Changes in muscle area from pre-to post program were calculated. A two-way repeated measures ANOVA was used to compare the effects between groups (HI vs. MIX) and times (Pre vs. Post). Furthermore, the sums of muscle thickness and fascia thickness of three regions were calculated, and One-way ANOVA was analysed to compare mean differences between groups in Pre and Post. Volume load was calculated using the formula: volume load = repetition (no.) × external load (Lbs.).

One-way ANOVA was used to compare differences in average volume load between groups in each week. Effect sizes were calculated using the following formula: meanchange/pooled SD, with interpretations based on conventional criteria of 0.00–0.19 was considered as *Trivial*, 0.20–0.49 as *Small*, 0.50–0.79 as *Moderate*, and ≥0.80 as *Large*. Statistical analyses were performed using IBM SPSS Statistics version 20, with a significance level set at α = 0.05.

## Results

Of the 20 participants initially recruited, two withdrew - one due to loss of interest and the other experiencing an unexpected injury. The final analysis included eight participants in the MIX group and ten in the HI group, with 100% adherence among those who completed the study. Statistical tests showed no significant differences at baseline (Table 1).

**Fig. 3:**
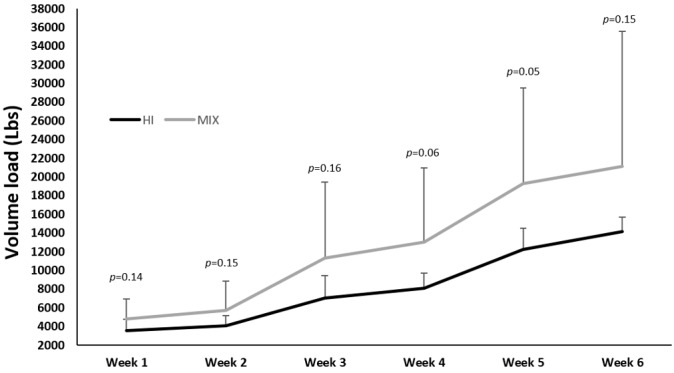
Mean and SD of average volume loads (formula: set x reps x loads) from week 1 to week 6. HI, high-intensity program; MIX, high-intensity with low-intensity blood flow restriction program

The average repetition numbers performed in high-intensity sets and low-intensity sets with practical BFR were 13.9 ± 4.5 repetitions and 46.2 ± 24.4 repetitions, respectively. Figure 3 displays the average volume load for each week. Although two different training programs yielded different volume loads each week, no statistical differences were observed between groups for any week (Figure 3).

A significant time effect was observed for the muscle area of the vastus lateralis at the proximal (F[1,16] = 17.34, p = 0.001, η_(2 )^p = 0.52), middle (F[1,16] = 19.78, p < 0.001, η_(2 )^p = 0.55), and distal regions (F[1,16] = 19.28, p < 0.001, η_(2 )^p = 0.55), with no significant main effect of group or interaction. Specifically, both the HI and MIX groups exhibited significant increases in muscle area at the proximal (Δ12%, p = 0.03, ES = Moderate and Δ9.2%, p < 0.001, ES = Small, respectively), middle (Δ8.7%, p = 0.02, ES = Moderate and Δ9.0%, p = 0.01, ES = Small, respectively), and distal (Δ14 %, p = 0.02, ES = Moderate and Δ13%, p = 0.002, ES = Moderate, respectively) regions as assessed by MRI. Further details are provided in Table 2.

**Fig. 4:**
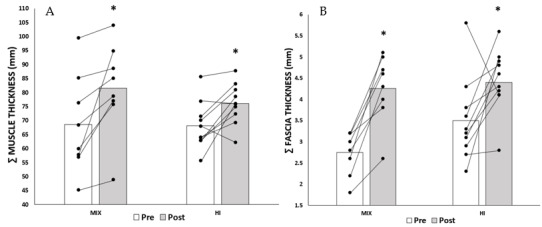
(A) Mean of sums of muscle thickness and (B) fascia thickness at Pre and Post with individual analysis in black dot. * p < 0.05 compared to Pre. Pre, pre-intervention; Post, post-intervention; MIX, high intensity with Low-intensity blood flow restriction program; HI, high-intensity program

Considering the sum of muscle thickness (Figure 4A), both the HI and MIX groups showed statistically significant increases in the sum of muscle thickness at Post, with Δ7.9 ± 7.9mm (p = 0.01, ES = Large) and Δ12.9±12.1mm (p = 0.02, ES = *Moderate*). Nevertheless, no statistically significant difference between groups was found both at Pre (F_[1,16]_ = 0.01, p = 0.94) and Post (F_[1,16]_ = 0.92, p = 0.35).

Similarly, significant increases in the sum of fascia thickness (Figure 4B) were observed in both HI and MIX groups following the training interventions, with Δ0.9±1.2mm (p = 0.04, ES = *Large*) and Δ1.5±0.6mm (p < 0.001, ES = *Large*), respectively. Nevertheless, no significant difference between groups at both time points was observed.

## Discussion

This study's main findings demonstrated that the HI and MIX programs effectively induced desirable increases in muscle area and sums of muscle and fascia thickness of vastus lateralis following the six-week training program. These findings aligned with previous findings in resistance training literature, indicating that both traditional high and low intensity with BFR resistance training effectively induced the vastus lateralis hypertrophy.[4] Nevertheless, it was difficult to directly compare our findings with the literature because of the absence of prior studies which never employed combined intensity programs on morphological adaptations.

Previous studies primarily compared high-intensity training to low-intensity training with BFR, rather than investigating the combined approach as our study did. Yasuda and colleagues conducted a notable attempt at this combination, where participants alternated low-intensity BFR training on Monday and Wednesday with high-intensity training on Friday. After 6 weeks, the pectoralis major muscle area increased by 10.5% with combined training, compared to 17.6% with high-intensity training alone and 8.3% with low-intensity training alone.[14] While the combined approach did not exceed the hypertrophic gains of high-intensity training, it did outperform low-intensity training. However, direct comparison was limited here as the study did not assess hypertrophic effects on the vastus lateralis muscle.

Additionally, Ellefsen and teams compared the hypertrophic effects of high-intensity (70% 1RM) and low-intensity (30% 1RM) BFR training using knee extensions in a within-subject design. They found significant increases in the vastus lateralis muscle area, with approximately 8% growth in the proximal region for high intensity and 7% for low intensity. Additionally, significant non-uniform hypertrophy was observed in the distal region, with increases of up to 7% and 10%, respectively.[4] Our current study partially supported these findings, showing that knee extension exercises resulted in non-uniform hypertrophy. We observed a significant increase in the distal muscle area of up to 12–14%, compared to 9–11% in the proximal area. Interestingly, the effect size for HI was larger than for MIX in proximal and middle muscle areas. This suggested that proximal and middle hypertrophy of the vastus lateralis might be more effectively stimulated by exclusively high-intensity training. Mechanistically, a previous study showed that higher muscle activation had been observed with high loads compared to low loads training to failure [15], suggesting that in the MIX group, the lower intensity sets might have led to suboptimal motor unit recruitment, resulting in less mechanical tension which was the key stimulus for muscle growth.[3]

It was noteworthy that, despite the different training program constructions between the HI and MIX, both groups exhibited a similar non-uniform pattern of hypertrophy. This suggested that leg extension exercises, regardless of the training protocol, might not have effectively promoted regional hypertrophy in every area. Other exercises might be needed to optimise regional growth. Therefore, from a practical standpoint, the findings from both HI and MIX supported the thought process of the hypertrophy program design, which is that practitioners aiming to maximise muscle growth across all muscle regions should incorporate multiple exercises targeting the muscle. Our results revealed that exclusively performing knee extension, either with HI or MIX program design, may not equally increase the size of every region of the vastus lateralis. Our findings were supported by previous studies demonstrating that performing leg press, hack squat, and half squat across training programs resulted in superior increases in the percentage of muscle regional hypertrophy across proximal, middle, and distal regions of vastus lateralis compared to doing leg press alone.[16]

Moreover, this finding of regional non-uniform hypertrophy aligned with a previous study by Martin-Hernandez and colleagues, which compared high-intensity (75% 1RM) to low-intensity BFR (20% 1RM) knee extension exercises. After 5 weeks, both groups showed a significant increase in muscle thickness of about 9.9%.[17] Similarly, our study found that vastus lateralis thickness increased by 12% (p = 0.01) in the high-intensity group and 19% (p = 0.02) in the mixed-intensity group. However, unlike their study, which measured muscle thickness only at the midpoint of the femur, this single random region might not have accurately reflected the overall adaptations resulting from the training [13], our study measured muscle thickness across three regions. This provided a more comprehensive view of exercise-induced hypertrophy, highlighting the importance of assessing multiple sites to capture a realistic picture of size adaptation. In addition to the significant increases in muscle area and thickness, our current findings also revealed a statistically significant increase in fascia thickness in both groups (p < 0.05). It was previously hypothesised that thick connective tissue might impede skeletal muscle growth.[18] However, our present study provided findings that challenge this hypothesis. Despite the significant increases in fascia thickness, concurrent muscle morphological adaptations were evident in both HI and MIX. Moreover, cells within the fascia layer, such as satellite cells, also played a pivotal role in muscle repair and regeneration.[3] Therefore, we proposed that the process of fascia remodelling might be linked to satellite cell function or the adaptive capacity of skeletal muscle.

Furthermore, it was worth discussing the impact of volume load on morphological adaptation. Previous research had suggested that increased volume load, such as adding more sets or reps to an exercise regimen, ultimately leads to increased muscle morphological adaptation. For instance, a previous study compared quadriceps muscle area following knee extensor training with either long or short rest intervals. They found that muscle morphological adaptation was equal between the groups when the short rest interval group performed additional repetitions to match the volume load of the long rest condition. However, if the short rest condition did not include additional repetitions, muscle morphological adaptation was inferior to that of the long rest condition.[19] These results aligned with the findings in previous meta-analysis suggesting that each additional set of training, which resulted in increased volume load, corresponded to a 0.37% increase in the percentage of muscle morphological adaptation.[20]

Our findings raised questions about volume load, as although the MIX group trained with a higher absolute volume load (75129lbs vs. 50062lbs), there was no difference in muscle hypertrophy between the groups. This suggested that increasing the total load did not necessarily lead to greater hypertrophy. Instead, our study, which equalized the number of weekly sets across programs, indicated that the volume set performed to failure might be more closely related to muscle growth. This aligned with a study by Kikuchi and Nakazato, where similar hypertrophy was achieved with loaded bench pressing and bodyweight push-ups to failure, given equal sets.[5] We proposed that sets to failure dictated muscle growth regardless of repetition range. Moreover, mechanically, previous studies suggest a dose-response relationship between training volume and muscle protein synthesis.[21] Although our study did not explore cellular signaling, it was probable that both groups reached a ceiling effect in muscle protein synthesis, thus resulting in similar hypertrophy. However, future research should examine cellular signaling in muscle protein synthesis from our training programs to confirm whether a ceiling effect contributes to the observed hypertrophy.

## Conclusion

In summary, our study revealed the effectiveness of both HI and MIX programs in eliciting significant muscle hypertrophy, as shown by increased muscle area and thickness. Moreover, despite variations in average weekly volume load between groups, both approaches yielded comparable outcomes in muscle hypertrophy. This suggested that the number of sets to failure could be a viable metric for quantifying training volume for hypertrophy purposes. Individuals may choose their preferred training program based on personal preferences or specific training goals.

## Figures and Tables

**Table 1:** Baseline of participants’ anthropometric and morphological characteristics

	HI (n = 10)	MIX (n = 8)	F	p-value

Age (years)	21.3 ± 0.7	21 ± 0.0	1.27	0.28
Height (cm)	173.1 ± 5.5	174.9 ± 5.3	0.48	0.50
Body mass (kg)	68.5 ± 11.4	69.0 ± 11.9	0.01	0.93
VL_proximal_ (mm^2^)	2489 ± 382	2782 ± 794	1.06	0.32
VL_middle_ (mm^2^)	2562 ± 377	2705 ± 629	0.36	0.56
VL_distal_ (mm^2^)	1587 ± 296	1696 ± 384	0.46	0.51
Sum of muscle thickness (mm)	68.1 ± 8.5	68.6 ± 17.9	0.01	0.94
Sum of fascia thickness (mm)	3.5 ± 1.0	2.8 ± 0.5	3.75	0.07
1RM knee extension (Lbs)	142.0 ± 19.9	153.8 ± 40.0	0.67	0.43

Data are represented as mean ± SD. HI, high intensity program; MIX, high intensity with low intensity blood flow restriction program; VL, vastus literalis muscle; 1RM, 1 repetition maximum

**Table 2:** Changes in muscle area (mm^2^) of vastus lateralis at proximal, middle, and distal regions

	HI (n=10)				MIX (n=8)				p-value		
	Pre	Post	Change (95%CI)	ES	Pre	Post	Change (95%CI)	ES	Group	Time	Interaction
VL_proximal_	2489 ± 382	2775 ± 598*	286 (37.3; 535)	0.57	2782 ± 794	3038 ± 797*	256 (148; 363)	0.32	0.37	0.001	0.82
VL_middle_	2562 ± 377	2785 ± 361*	223 (62.5; 382)	0.60	2705 ± 629	2950 ± 633*	245 (62.0; 429)	0.39	0.52	0.000	0.88
VL_distal_	1587 ± 296	1810 ± 349*	223 (40.8; 404)	0.69	1696 ± 384	1911 ± 403*	215 (112; 318)	0.55	0.53	0.000	0.94

Data are represented as mean ± SD.

* p < 0.05 comapared to Pre. 95%CI, 95% confidence interval; HI, high intensity program; MIX, high intensity with Low intensity blood flow restriction program; VL, vastus literalis muscle; ES, effect size
